# The First Complete Chloroplast Genome Sequence of *Secale strictum* subsp. *africanum* Stapf (*Poaceae*), the Putative Ancestor of the Genus *Secale*

**DOI:** 10.3390/cimb47010064

**Published:** 2025-01-17

**Authors:** Lidia Skuza, Piotr Androsiuk, Romain Gastineau, Magdalena Achrem, Łukasz Paukszto, Jan Paweł Jastrzębski

**Affiliations:** 1 Institute of Biology, University of Szczecin, PL-71-415 Szczecin, Poland; 2 Centre for Molecular Biology and Biotechnology, University of Szczecin, PL-71-415 Szczecin, Poland; 3 Department of Plant Physiology, Genetics and Biotechnology, Faculty of Biology and Biotechnology, University of Warmia and Mazury, PL-10-719 Olsztyn, Poland; piotr.androsiuk@uwm.edu.pl (P.A.);; 4 Institute of Marine and Environmental Sciences, University of Szczecin, PL-70-383 Szczecin, Poland; romain.gastineau@usz.edu.pl; 5 Department of Botany and Evolutionary Ecology, Faculty of Biology and Biotechnology, University of Warmia and Mazury in Olsztyn, PL-10-721 Olsztyn, Poland

**Keywords:** *Secale strictum* ssp. *africanum* (*Poaceae*), chloroplast, weedy rye, phylogenetic analysis

## Abstract

*Secale strictum* ssp. *africanum* (synonym *Secale africanum*), a putative ancestor of the genus *Secale*, has been classified within *Secale strictum*, although recent phylogenetic studies suggest that it represents a distinct species. This study reports the first complete chloroplast genome of *S. africanum*, highlighting its structure, genetic composition, and phylogenetic relationships within *Secale* and related Triticiceae species. Phylogeny reconstruction based on the maximum-likelihood method reveals notable genetic similarity between *S. strictum* and *S. africanum*, supporting their genetic and phylogenetic distinction. Here, we assembled the complete, annotated chloroplast genome sequence of *Secale strictum* ssp. *africanum*. The genome is 137,068 base pair (bp) long. It is the first complete chloroplast genome that can be used as a reference genome for further analysis. The genome can be accessed on GenBank with the accession number OQ700974. This work sheds light on the evolutionary history of *Secale* and contributes to our understanding of chloroplast genomics in cereal ancestors, with potential applications in improving cereal crop resilience, advancing breeding strategies, and informing conservation efforts for genetic diversity.

## 1. Introduction

*Secale strictum* ssp. *africanum* Stapf (*Poaceae*) (synonym *Secale africanum*) is recognized as one of the oldest lineages within the genus *Secale* [1]. Despite its long-standing taxonomic significance, the exact classification of *S. africanum* has been subject to extensive debate due to its morphological and genetic resemblances with *S. strictum* [2]. Early studies, particularly by Hammer [3,4], suggested a close relationship between *S. africanum* and *S. strictum*, often grouping them within the same species complex. However, recent advances in genetic analysis, particularly through single-nucleotide polymorphism (SNP) data [5], have provided evidence of sufficient divergence to consider *S. africanum* a distinct species. Unlike other species in the genus, *S. africanum* is endemic to southern Africa [6] and displays the ability to hybridize with other *Secale* species, an occurrence which suggests potential historical human-mediated dispersal [7].

The unique genetic and evolutionary characteristics of wild *Secale* species, including *S. africanum*, hold significant potential in agricultural science. Wild species are invaluable resources in breeding programs, where they are used to obtain distant hybrids with cultivated rye. Such crossbreeding can expand recombinant variability and allows for observations of growth and development of recombinants in heterotic, transgressive, and population-based breeding. The interaction of genomes from different species or subspecies enables the expression of genes with additive and epistatic effects, determining qualitative and quantitative traits [8]. As SNP-based phylogenies are considered to be the most accurate methods of phylogeny reconstruction [9], SNPs identified and extracted from complete chloroplast genome sequences of *S. africanum* and closely related species have been applied in evolutionary analyses.

This study aims to further clarify the taxonomic placement of *S. africanum* through chloroplast (cp) genome analysis, which may provide insights into its evolutionary history and genetic divergence within the *Secale* genus. These findings could have broader implications for understanding the genetic diversity and evolutionary adaptations within this genus, supporting conservation strategies and facilitating the use of *S. africanum* and related species in crop improvement and breeding programs.

## 2. Materials and Methods

Seeds of *Secale strictum* ssp. *africanum* (introd. no. 6043) were obtained from the Plant Breeding and Acclimatization Institute National Centre for Plant Genetic Resources, Poland. Total DNA was extracted from young sprouts following Doyle and Doyle [10].

The cp genome of *S. africanum* was sequenced with the use of the DNBseq platform in BGI Shenzhen (Shenzhen, China). After the quality check (FastQC v.0.11.9 tool; Babraham Institute, Cambridge, UK; available online at http://www.bioinformatics.babraham.ac.uk/projects/fastqc), the raw reads were mapped to the reference genome of *Secale cereale* (NC_021761) [11] in the Geneious v.R7 (Biomatters, Auckland, New Zealand; accessed on 10 June 2022) software with default medium–low sensitivity settings [12]. Reads aligned to the reference cpDNA genome were extracted and used for de novo assembly (K-mer, 23–41; low-coverage cut-off, 5; and minimum contig length, 300; the parameters values were determined experimentally and positively verified in the assembly of the organellar genomes [13,14]).

De novo contigs were extended by mapping raw reads to the generated contigs, reassembling the contigs with mapped reads, and manually scaffolding the extended contigs (minimum sequence overlap of 50 bp and 97% overlap identity). This process was iterated five times. Finally, the reduced sequences were assembled in the circular chloroplast genome. The cp genome was annotated using MFannot v.1.1. (Centre Robert-Cedergren Bio-Informatique et Genomique, University of Montreal, Montreal, QC, Canada) [15] and PlasMapper 2.0 (available online: https://plasmapper.wishartlab.com/; Wishart Laboratory at the University of Alberta, Canada; accessed on 15 June 2022) [16], with default settings. The gene map of the annotated cp genome was developed with the OrganellarGenome DRAW v.1.3.1 tool (available online https://chlorobox.mpimp-golm.mpg.de/OGDraw.html; Max Planck Institute of Molecular Plant Physiology, Potsdam-Golm, Germany; accessed on 4 August 2022) [17]. Finally, the phylogenetic position of *S. africanum* within the Triticodae group was also evaluated. A phylogenetic tree was constructed with the use of the maximum-likelihood (ML) method implemented in IQ-TREE v. 2.2.0.3 (Australian National University, Canberra, Australia) [18] with the GTR + F model selected by ModelFinder v.2 (The University of Hong Kong, Hong Kong) [19]. The ML analysis was performed with 1000 bootstrap replicates. The analysis was based on the alignment of SNP regions identified and extracted by the PhaME v.1.0.4 software (Los Alamos National Laboratory, Los Alamos, NM, USA) [20] from the complete cp genomes of *S. africanum* and selected representatives of Triticiceae. The option 3 (MinHash distance) of PhaME was used to determine the reference genome from a given set of genomes.

## 3. Results

**Figure 1:**
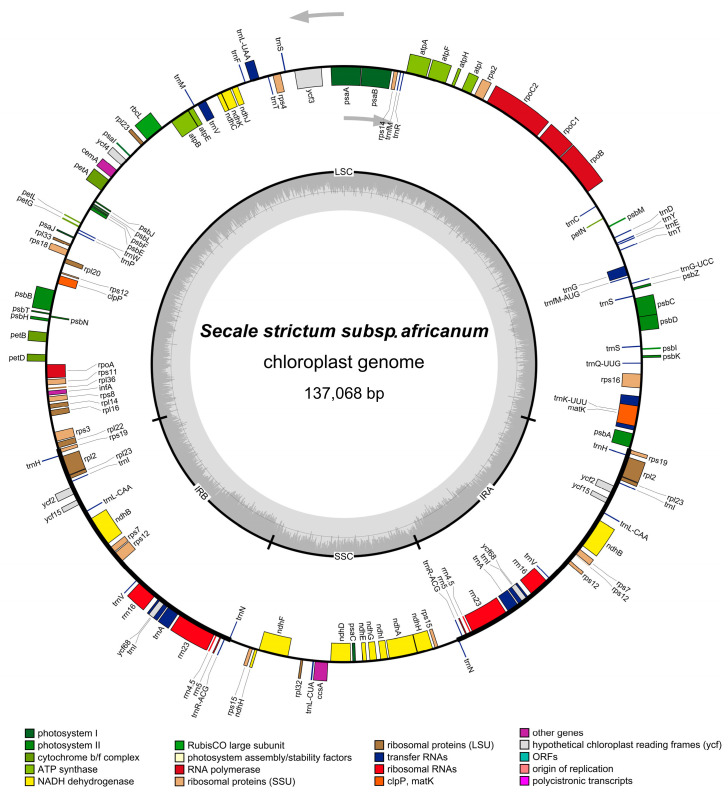
Map of the chloroplast genome of *Secale africanum*. The genes inside and outside the circle are transcribed in the clockwise and counterclockwise directions, respectively. Genes belonging to different functional groups are shown in different colors. The thick lines indicate the extent of the inverted repeats (IRa and IRb) that separate the genomes into small single-copy (SSC) and large single-copy (LSC) regions. The innermost darker gray corresponds to the GC content while the lighter gray corresponds to the AT content.

The *S. africanum* cp genome appears as a typical circular, double-stranded molecule with a length of 137,068 bp (GenBank: OQ700974) and an overall GC content of 38.23% (Figure 1). The large single-copy (LSC) region is 81,080 bp long, the short single-copy (SSC) region is 12,818 bp long, and each of the inverted repeat regions (IRs) is 21,585 bp long. The reported cp genome contains 137 genes, including 113 unique genes and 24 genes which are duplicated in the IRs. The group of 113 unique genes features 73 protein-coding genes, 30 tRNA genes, 4 rRNA genes and 5 conserved chloroplast open reading frames (ORFs). The LSC region appears as the most abundant in genes—57 PCGs, 21 tRNA genes and 2 ORFs (ycf3 and ycf4)—whereas there are only 10 PCGs and 1 tRNA gene in the SSC. In the IR, there are four rRNA genes, eight tRNA genes, three ORFs (ycf2, ycf15, and ycf68), and nine PCGs, including ndhH, located on the junction between the IR and the SSC region (Figures S1–S4).

**Figure 2:**
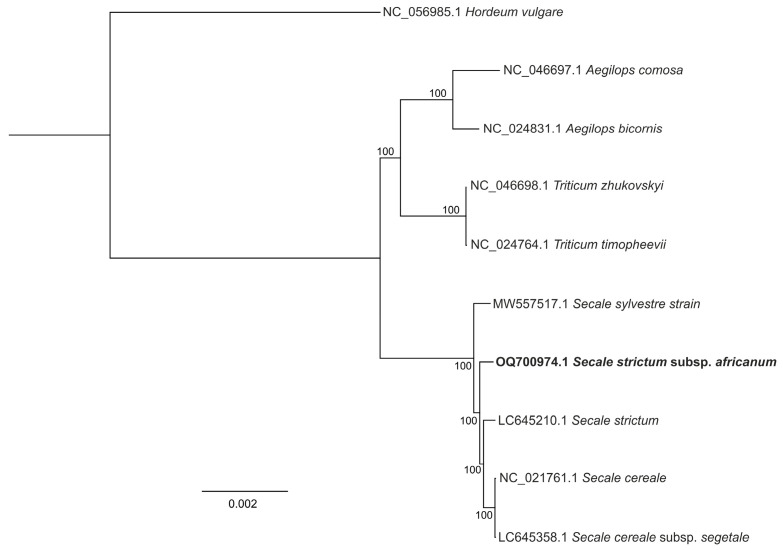
Cladogram illustrating the phylogenetic relationships of *Secale africanum* based on complete cp genome sequences, using the maximum-likelihood method implemented in IQ-TREE with the GTR + F model selected by ModelFinder. The cpDNA sequence obtained in this study is shown in bold.

Phylogenetic reconstruction revealed that *S. africanum* is close to the other Secale cp genomes (Figure 2). Overall, our study provides valuable genetic data for phylogenetic and evolutionary studies of the genus Secale.

## 4. Discussion

Hybrid rye varieties have advanced breeding by enabling genotype fixation and trait transfer, improving yields but with fluctuating intermediate quality traits [21,22].

Wild *Secale* species, such as *S. strictum* and *S. vavilovii*, offer valuable genetic diversity, including disease resistance and sterilizing cytoplasm, making them important for enhancing variability in rye and triticale breeding [23]. However, limited phylogenetic knowledge slows progress.

Chloroplast genomes, with their small size and slow mutation rate, are crucial for phylogenetic and genetic studies. While largely conserved, structural variations such as gene rearrangements have been observed across families and genera (e.g., [24]).

In this study, the cp genome of *S. africanum* (Figure S5) was newly assembled and annotated. Its chloroplast genome length, GC content, and gene composition were similar to previously sequenced plastomes of *S. cereale* (137,051 bp) [11], *S. sylvestre* (137,116 bp) [25], and *S. segetale* (137,042 bp) [14], within the size range of angiosperms [26].

This phylogenetic analysis supports the close relationship between *S. africanum* and *S. strictum*.

This study reinforces the hypothesis that the restricted distribution of *S. africanum* in southern Africa may be the result of historical human dispersal rather than an ancient, widespread distribution. Its genetic similarity to *S. strictum* aligns with theories proposing that the distribution of *Secale* was influenced by early agricultural activities, possibly explaining why *S. africanum* is found so far from other *Secale* taxa.

This work provides the first complete chloroplast genome sequence for *Secale strictum* ssp. *africanum* and contributes valuable information regarding its genetic and evolutionary relationship with *S. strictum*. High genetic similarity, despite geographical isolation, supports the classification of *S. africanum* as a separate species, while hinting at human activities shaping its current distribution. The data presented here enhance our understanding of *Secale* evolution and highlight the role of chloroplast genomics in clarifying relationships within cereal ancestors.

However, this study is limited by its focus on chloroplast genomes, which, while informative, provide only part of the picture of genetic divergence and evolutionary history. Future research could integrate nuclear and mitochondrial genome analyses to obtain a more comprehensive understanding of the genetic structure and evolutionary dynamics of *S. africanum*. Additionally, exploring ecological and environmental factors influencing its restricted distribution could offer insights into its adaptive traits and potential resilience under changing climatic conditions.

## Data Availability

The genomic sequence data that support the findings of this study are openly available in the GenBank of NCBI (https://www.ncbi.nlm.nih.gov/search/all/?term=OQ700974) under accession number OQ700974 (accessed on 12 January 2025).

## Supplementary Materials

The following supporting information can be downloaded at https://www.mdpi.com/article/10.3390/cimb47010064/s1.
