# Supplementary material for: The First Complete Chloroplast Genome Sequence of Secale strictum subsp. africanum Stapf (Poaceae), the Putative Ancestor of the Genus Secale
Source: Curr Issues Mol Biol. 2025 Jan 17;47(1):64. doi: 10.3390/cimb47010064 (PMC11764287; doi:10.3390/cimb47010064)
Supplement: Supplementary file 1 [file cimb-47-00064-s001.zip › figure captions.pdf]

Figure S1: Read coverage plot for sequenced chloroplast genome of *Secale strictum* subsp. *africanum* Stapf. This plot displays the depth of sequencing coverage (blue histogram) across the entire chloroplast genome and plastid genes annotation (yellow - CDSs, green - genes, red - rRNAs, pink - tRNAs);
Figure S2: Exon-intron structure of cis-splicing genes from chloroplast genome of *Secale strictum* subsp. *africanum* Stapf;
Figure S3: Schematic representation of the trans-spliced rps12 gene structure from the chloroplast genome of *Secale strictum* subsp. *africanum* Stapf. This gene comprises three unique exons, with two of them duplicated and situated in the inverted repeat (IR) regions. The yellow graph illustrates the rps12 coding sequence (CDS) structure when all three exons are positioned on the complementary strand. The orange graph depicts a mixed rps12 CDS structure, where one exon is located on the complementary strand and the other two are found on the forward strand, oriented oppositely due to their placement within the IR region;
Figure S4: Exon-intron structure of all genes annotated in chloroplast genome of *Secale strictum* subsp. *africanum* Stapf.
Figure S5: The seeds of *S. africanum*.
