# Supplementary material for: The First Complete Chloroplast Genome Sequence of Secale strictum subsp. africanum Stapf (Poaceae), the Putative Ancestor of the Genus Secale
Source: Curr Issues Mol Biol. 2025 Jan 17;47(1):64. doi: 10.3390/cimb47010064 (PMC11764287; doi:10.3390/cimb47010064)
Supplement: Supplementary file 1 [file cimb-47-00064-s001.zip › Figure S2.pdf]

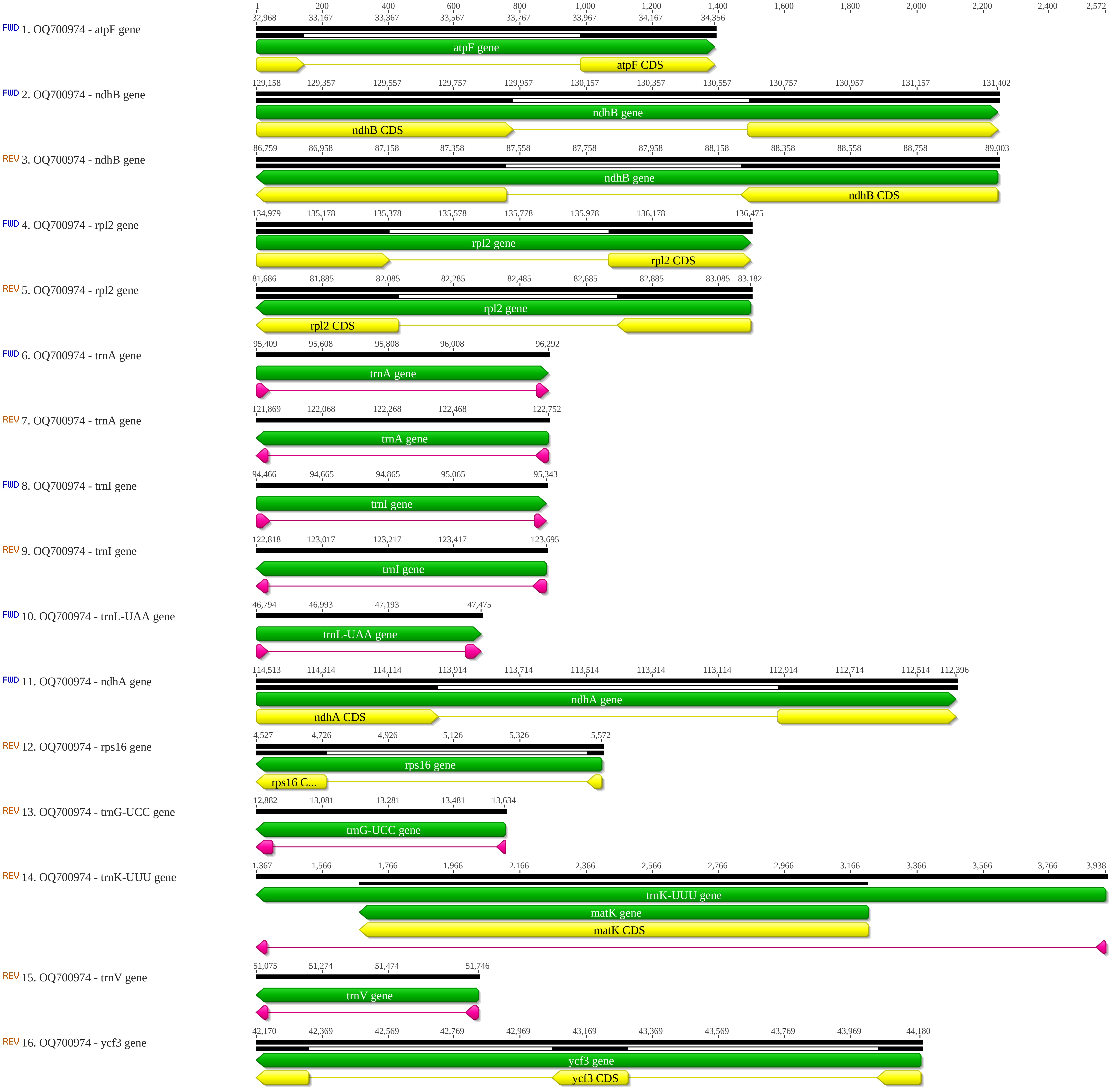
1 200 400 600 800 1,000 1,200 1,400 1,600 1,800 2,000 2,200 2,400 2,572
FWD 1. OQ700974 - atpF gene
32,968 33,167 33,367 33,567 33,767 33,967 34,167 34,356
atpF gene
atpF CDS
FWD 2. OQ700974 - ndhB gene
129,158 129,357 129,557 129,757 129,957 130,157 130,357 130,557 130,757 130,957 131,157 131,402
ndhB gene
ndhB CDS
REV 3. OQ700974 - ndhB gene
86,759 86,958 87,158 87,358 87,558 87,758 87,958 88,158 88,358 88,558 88,758 89,003
ndhB gene
ndhB CDS
FWD 4. OQ700974 - rpl2 gene
134,979 135,178 135,378 135,578 135,778 135,978 136,178 136,475
rpl2 gene
rpl2 CDS
REV 5. OQ700974 - rpl2 gene
81,686 81,885 82,085 82,285 82,485 82,685 82,885 83,085 83,182
rpl2 gene
rpl2 CDS
FWD 6. OQ700974 - trnA gene
95,409 95,608 95,808 96,008 96,292
trnA gene
REV 7. OQ700974 - trnA gene
121,869 122,068 122,268 122,468 122,752
trnA gene
FWD 8. OQ700974 - trnI gene
94,466 94,665 94,865 95,065 95,343
trnI gene
REV 9. OQ700974 - trnI gene
122,818 123,017 123,217 123,417 123,695
trnI gene
FWD 10. OQ700974 - trnL-UAA gene
46,794 46,993 47,193 47,475
trnL-UAA gene
FWD 11. OQ700974 - ndhA gene
114,513 114,314 114,114 113,914 113,714 113,514 113,314 113,114 112,914 112,714 112,514 112,396
ndhA gene
ndhA CDS
REV 12. OQ700974 - rps16 gene
4,527 4,726 4,926 5,126 5,326 5,572
rps16 gene
rps16 C...
REV 13. OQ700974 - trnG-UCC gene
12,882 13,081 13,281 13,481 13,634
trnG-UCC gene
REV 14. OQ700974 - trnK-UUU gene
1,367 1,566 1,766 1,966 2,166 2,366 2,566 2,766 2,966 3,166 3,366 3,566 3,766 3,938
trnK-UUU gene
matK gene
matK CDS
REV 15. OQ700974 - trnV gene
51,075 51,274 51,474 51,746
trnV gene
REV 16. OQ700974 - ycf3 gene
42,170 42,369 42,569 42,769 42,969 43,169 43,369 43,569 43,769 43,969 44,180
ycf3 gene
ycf3 CDS
