# Supplementary figures and images for: The First Complete Chloroplast Genome Sequence of Secale strictum subsp. africanum Stapf (Poaceae), the Putative Ancestor of the Genus Secale
Source: Curr Issues Mol Biol. 2025 Jan 17;47(1):64. doi: 10.3390/cimb47010064 (PMC11764287; doi:10.3390/cimb47010064)

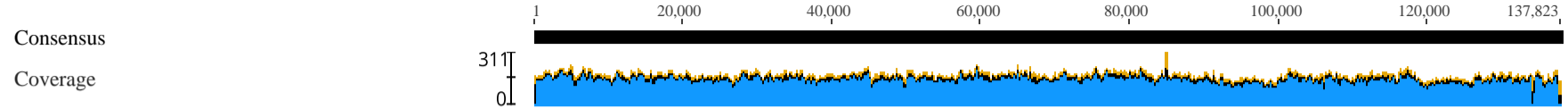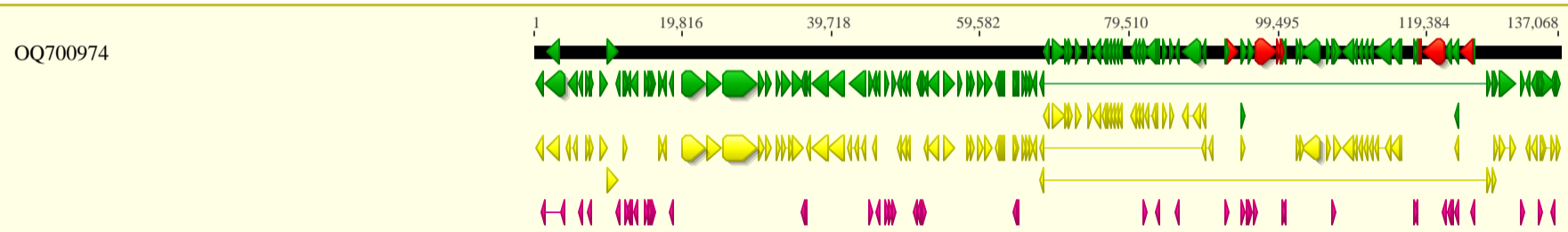

Supplement: Supplementary file 1 [file cimb-47-00064-s001.zip › Figure S1.pdf]

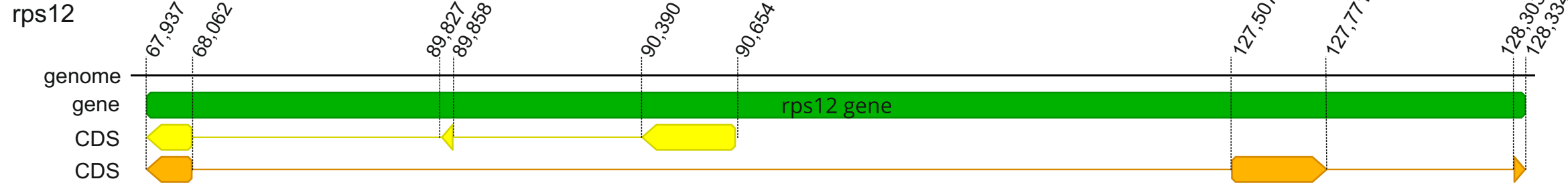

Supplement: Supplementary file 1 [file cimb-47-00064-s001.zip › Figure S3.pdf]

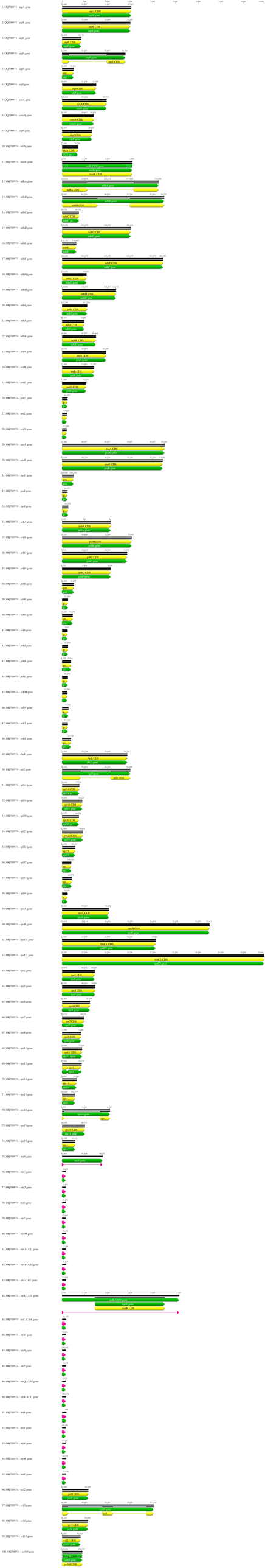

Supplement: Supplementary file 1 [file cimb-47-00064-s001.zip › Figure S4.pdf]

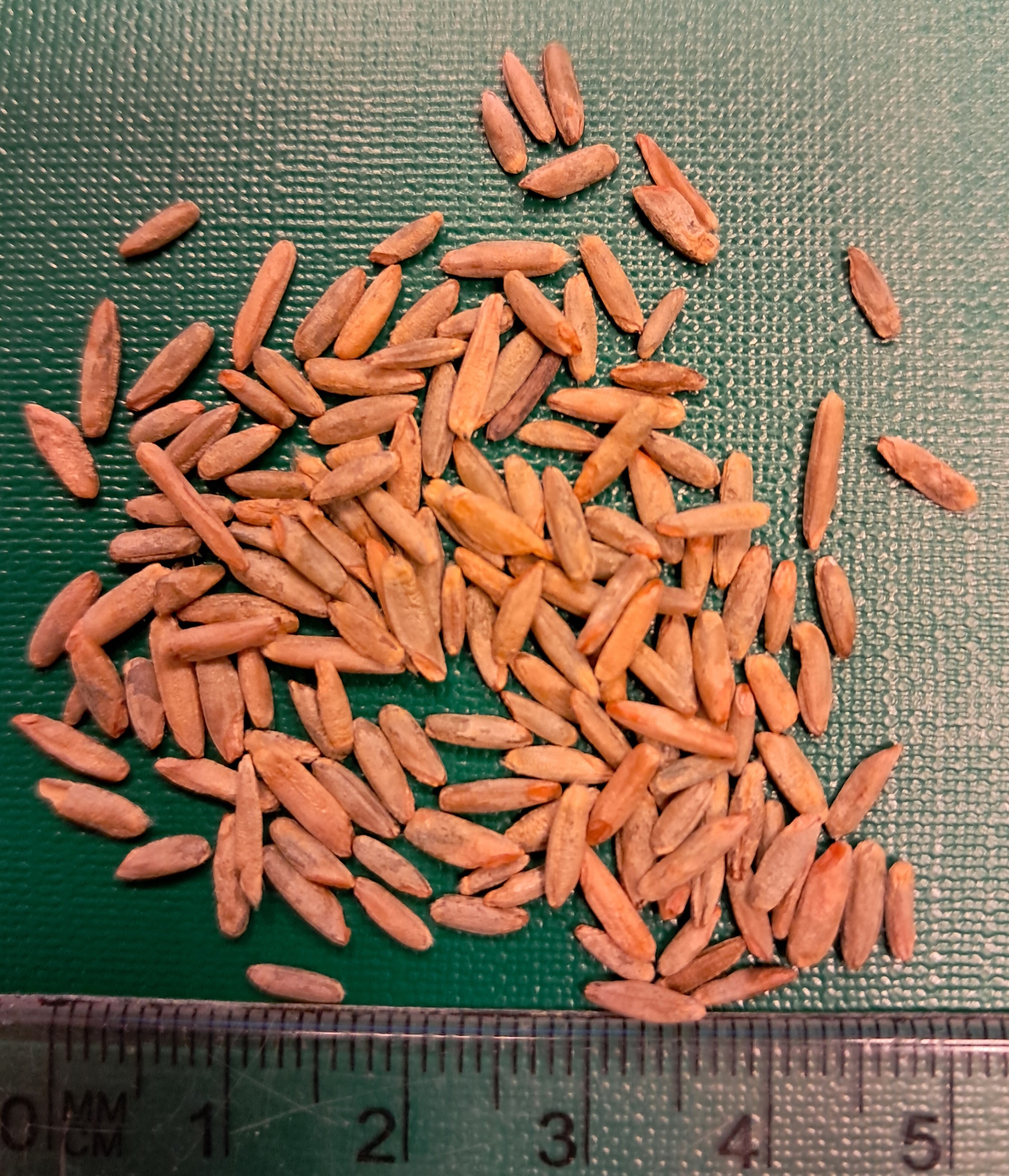

Supplement: Supplementary file 1 [file cimb-47-00064-s001.zip › Figure S5.jpg]
